# Cooperative Effects of Interface Symmetry, Redox Conditions and Low-Thickness to Improve Polarization in Ferroelectric Hf_0.5_Zr_0.5_O_2_ Films

**DOI:** 10.1021/acsami.5c03527

**Published:** 2025-05-26

**Authors:** Xueliang Lyu, Faizan Ali, Tingfeng Song, Ignasi Fina, Florencio Sánchez

**Affiliations:** Institut de Ciència de Materials de Barcelona (ICMAB-CSIC), Campus UAB, 08193 Bellaterra, Barcelona, Spain

**Keywords:** ferroelectric oxides, ferroelectric hafnia, hafnium oxide, Hf_0.5_Zr_0.5_O_2_, epitaxial films

## Abstract

The ferroelectric phase of hafnia is metastable, and its stabilization is achieved by appropriate doping and generally only in ultrathin films where the contribution of surface energy is relevant. Other factors, such as interfaces and point defects such as oxygen vacancies, can affect the formation energy of competing polymorphs. Understanding the role of these factors is important to achieve further control over the stabilized phases and, thereby, improve ferroelectric polarization. To gain insight into the role of defects and stress at interfaces, we have compared a series of Hf_0.5_Zr_0.5_O_2_ epitaxial films of various thicknesses. The films were grown on (001) and (110) oriented SrTiO_3_ substrates to impose different symmetries at the interface and were deposited in a pure O_2_ or a mixed O_2_/Ar atmosphere to vary the oxidation conditions. We find that both factors are critical, with polarization maximized in films on (110)-oriented substrates and prepared under reducing conditions. Irrespective of the used substrate and atmosphere, polarization rapidly decays for thicknesses above 10 nm, indicating the relevance of the surface energy. Strain is thickness dependent, varying differently depending on the substrate orientation, but not on the deposition conditions investigated. Strain-thickness and polarization-thickness dependencies are not correlated, signaling that strain does not have a direct influence on the ferroelectricity of the films. Thickness, oxidation conditions, and epitaxial stress can contribute synergistically, and films with an optimal selection of these parameters have the ferroelectric polarization expected for pure orthorhombic phase films.

## Graphical abstract

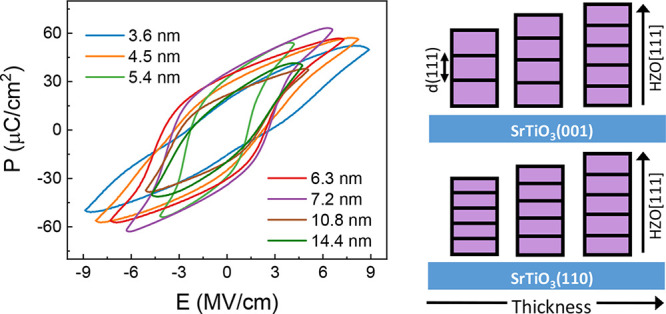

## 1 Introduction

The discovery of ferroelectricity in nanometric thin films of doped HfO_2_ has generated great interest due to the CMOS compatibility of this material.^1^ Despite the enormous potential of ferroelectric hafnia for memories, the progress needed for its use in commercial devices is limited by the complexity of the material.^2^ Ferroelectricity in nanometric HfO_2_ films arises in a metastable orthorhombic phase, which generally coexists with other polymorphs, and the films present extended defects such as boundaries between grains of different polymorphs or between grains with different crystalline orientations.^3–8^ In addition to doping with Zr or other cations, several factors such as stress and strain, oxygen vacancies, and other point defects can have a crucial impact on the formation of the orthorhombic phase.^2,9–15^ Oxygen vacancies are particularly critical. Materano et al.^16^ reported the coexistence of tetragonal and orthorhombic phases in Hf_0.5_Zr_0.5_O_2_ (HZO) films prepared by atomic layer deposition (ALD) when the ozone dose time was short. The orthorhombic phase was the majority with increasing ozone dose time, although for long times, the main phase was the monoclinic one. A similar dependence was reported for polycrystalline films prepared by sputtering. Low and high oxygen fluxes favored the formation of the tetragonal and monoclinic phase, respectively, while the ferroelectric orthorhombic phase was stabilized at an optimal intermediate oxygen flux.^17,18^ Low oxygen partial pressure in pulsed laser deposition (PLD) was also reported to be beneficial for epitaxial stabilization of the orthorhombic phase.^19^ On the contrary, discrepant conclusions have been reported in the literature regarding the influence of strain. Xu et al.^13^ investigated the structural and ferroelectric properties of a series of ZrO_2_ films prepared by ALD and chemical solution deposition. They observed that a large in-plane tensile strain stabilizes the tetragonal phase and a small in-plane tensile strain favors the monoclinic phase, while the orthorhombic ferroelectric phase is stabilized at an intermediate strain. Based on these findings, they concluded that there is a general relationship between strain and phase formation. On the other hand, Song et al.^12^ analyzed the orthorhombic phase content, strain, and polarization in various series (varying deposition parameters, dopant, and substrates) of doped HfO_2_ epitaxial films, and concluded that stress was critical to stabilize the ferroelectric phase, and ruled out a direct main influence of strain. Therefore, the role of strain in stabilizing the orthorhombic phase is a matter of debate.

Epitaxial growth of ferroelectric hafnia was first reported in 2016, by pulsed laser deposition and using fluorite single crystals of yttria-stabilized zirconia as substrate.^20,21^ Two years later, in 2018, epitaxial growth of (111)-oriented hafnia films on perovskite substrates with a (001)-oriented La_0.67_Sr_0.33_MnO_3_ (LSMO) bottom electrode was demonstrated.^22–24^ The use of perovskite substrates and LSMO electrode has allowed us to achieve excellent properties, considering ferroelectric polarization and reliability. Different strategies have made it possible to minimize the fraction of nonferroelectric phases and thus increase polarization. As mentioned above, epitaxial growth by PLD under a low partial pressure of oxygen (*P*_O2_) to favor the presence of oxygen vacancies has allowed an increase in ferroelectric polarization.^19,25^ On the other hand, stress engineering proved to be an appropriate strategy.^26^ In particular, scandate substrates, with a larger lattice parameter than the commonly used SrTiO_3_ (STO) substrate, induce tensile strain on the LSMO electrodes, and HZO films on these substrates present a less monoclinic phase. More recently, LSMO(110) electrodes on STO(110)^27,28^ or (110)-oriented (LaAlO_3_)_0.3_-(Sr_2_AlTaO_6_)_0.7_ (LSAT)^29^ have been used in place of LSMO(001) on (001)-oriented substrates. Despite the different symmetry at the interface, the epitaxial hafnia films maintain the (111) orientation and show less parasitic monoclinic phase than the equivalent films on LSMO(001). Some authors have associated the improvement of the ferroelectric properties of films on these (110) oriented substrates to strain,^27,29,30^ and particularly to anisotropic strain^30,31^ or strain gradient, and a resulting flexoelectric effect.^29^ On the other hand, a huge remanent ferroelectric polarization of about 50 μC/cm^2^ has been reported in Y-doped HfO_2_(111) films on LSMO(110)/STO(110), and the high polarization was associated with the high crystal quality of the films, obtained under optimized deposition conditions.^32^ However, this huge polarization exceeds the expected value of about 32 μC/cm^2^ considering the (111) orientation and the usual calculations of intrinsic polarization of approximately 52–55 μC/cm^2^ along the *c*-axis of orthorhombic hafnia.^33,34^ The polarization is also lowered if the film contains a fraction of nonferroelectric monoclinic phase, or when part of the orthorhombic phase cannot be switched due to the presence of defects that pin polarization.

Overall, it is evident that the substrate lattice parameter, substrate orientation, and oxidation condition during thin film growth are critical in the crystallization of competing polymorphs and measured polarization. These factors, potentially relevant to the energy balance between hafnia phases, could perhaps combine to facilitate the formation of the orthorhombic phase, similar to how doping and epitaxial stress synergistically do.^35^ To evaluate the different effects of interface symmetry and oxidation conditions, we have performed a comprehensive study comparing three series of seven films with varied thicknesses. Series A and B were deposited under a pure oxygen atmosphere (*P*_O2_ = 0.1 mbar) on LSMO buffered STO(001) and STO(110) substrates, respectively, and series C was deposited under a mixed atmosphere of oxygen (*P*_O2_ = 0.05 mbar) and argon (*P*_Ar_ = 0.05 mbar) on STO(110). It is found that in all three series the polarization decreases strongly in films thicker than approximately 10 nm, demonstrating the importance of surface energy contribution. For thinner films, the polarization is greater in films on STO(110) than in films on STO(001), revealing the relevant role of interface symmetry and resulting stress, and there is a further enhancement in films deposited under low oxidation conditions, which evidence that vacancies help to stabilize the orthorhombic phase. The thickness dependence of the strain is opposite for films on STO(110) and films on STO(001), while the atmosphere used here (0.1 mbar of pure O_2_ in series A and B and a mixture of 0.05 mbar of Ar and 0.05 mbar of O_2_ in series C) to grow the films does not have a significant influence on the strain.^19^ We rule out important direct effects of strain on the polarization of ferroelectric hafnia.

**Figure 1:**
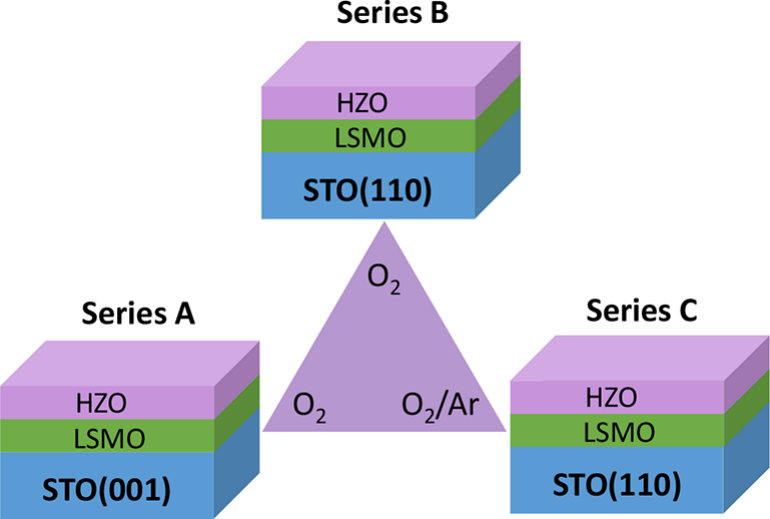
Sketch of the samples. Three series of seven HZO films of varied thickness were grown by PLD under high oxidation conditions (pure O_2_, series A and B) or low oxidation conditions (mixed O_2_/Ar, series C). HZO films of series A were deposited on LSMO/STO(001) and films of series B and C on LSMO/STO(110).

## 2 Experimental Section

Top HZO films and bottom LSMO electrodes were deposited in a single process by PLD on the STO(001) and STO(110) substrates. PLD was carried out with a KrF excimer laser (248 nm wavelength) operated at 5 Hz for LSMO and 2 Hz for HZO. The LSMO electrodes (thickness ∼ 25 nm) were grown at a substrate temperature of 700 °C under 0.1 mbar of oxygen. Two series of HZO films of varied thickness (in the 4.5–18.1 nm range) were deposited simultaneously on LSMO/STO(001) and LSMO/STO(110) substrates at 800 °C under pure O_2_ (0.1 mbar). The thickness of the films was controlled by changing the number of laser pulses in the 400–1600 pulses range, being a growth rate of 0.11 Å/pulse. A third series (varying the laser pulses in the 400–1600 pulses range) was grown on LSMO/STO(110) at the same temperature of 800 °C but under a mixed Ar (0.05 mbar)/O_2_ (0.05 mbar) atmosphere. The growth rate under this atmosphere was 0.09 Å/pulse, and the corresponding thickness range in the series is 3.6 to 14.4 nm. The samples of the three series were cooled under 0.2 mbar of oxygen. It should be noted that although the deposition atmosphere has an important influence on the crystallized phases and ferroelectric properties,^19^ the deposited films are highly stable and hardly sensitive to cooling conditions.^36^ The three series are sketched in Figure 1 and the thickness of all samples is indicated in Table S1.

Structural characterization was carried out by X-ray diffraction (XRD) using Cu Kα radiation, using diffractometers with either a point or area detector. Circular platinum top electrodes (thickness 20 nm and diameter 20 μm), deposited by DC magnetron sputtering through stencil masks, were used for measurement of ferroelectric polarization loops (dynamic leakage current compensation (DLCC) procedure, frequency of 1 kHz, top-bottom configuration grounding the bottom electrode, room temperature) using an AixACCT TFAnalyser2000 platform.

## 3 Results

Figure 2a shows XRD θ-2θ scans, measured with a point detector, of the HZO films deposited on STO(001) with a varied number of laser pulses under a pure O_2_ atmosphere (series A). For clarity, scans are vertically shifted by HZO thickness (*t*); the scan that corresponds to the thinnest film (*t* = 4.5 nm, violet line) at the bottom and the scan that corresponds to the thickest (18.1 nm, red line) at the top. The main peak in all films is at 2θ ≈ 30°, the position corresponding to the orthorhombic (o) o-(111), the tetragonal (110), and the cubic (111) reflections. However, the formation of cubic or tetragonal phases is not expected in epitaxial HZO films on (001) and (110)-oriented STO substrates. In particular, transmission electron microscopy characterization did not reveal the presence of these phases.^26,28,37,38^ We had only observed the presence of the tetragonal/cubic phase in epitaxial ZrO_2_ films.^39^ Therefore, the peak located at 2θ ≈ 30° is indexed as o-(111). With increasing thickness, the peak becomes narrower, with full-width at half-maximum (FWHM) values (Figure S1) decreasing from 1.78° (*t* = 4.5 nm) to 0.52° (*t* = 18.1 nm). Laue fringes around the o-(111) peak signal high crystalline quality, flat interfaces, and homogeneous thickness. The width of the o-(111) peak and the spacing of the fringes depend on the thickness, and simulation (Figure S2) was done to determine the thickness of HZO. The vertical dashed red line in Figure 2a marks the position of the o-(111) peak in the thickest film. It can be seen that the position of the peak changes progressively to higher angles with increasing thickness, revealing a progressive reduction of the out-of-plane lattice parameter, *d*_o‑(111)_. In addition to the o-(111) peak, a very low-intensity peak at approximately 34–35° can be seen in some films, indicating the presence of a small number of {100}-oriented monoclinic or orthorhombic crystallites. More evident is the peak at around 28.3° in the thicker films, which corresponds to the m-(−111) reflection. Further XRD measurements were performed using a two-dimensional detector. The selected 2θ-χ images in Figure 2a correspond to *t* = 4.5 nm (bottom), *t* = 9.0 nm (middle), and *t* = 18.1 nm (top) films (the 2θ-χ images of all films can be seen in Figure S3. The θ-2θ diffractograms on the right of each image in Figure 2a were obtained by integration in the range χ = ± 10°. In addition to the substrate and LSMO reflections, the o-(111) reflection is seen in the 2θ-χ images. The reflection is a spot at χ = 0°, and the circular rather than arc-like shape agrees with the epitaxial growth of HZO on (001) and (110)-oriented LSMO.^22,28^ Figure S4 shows χ curves of selected films and the FWHM thickness dependence of the χ curves for all films in the series. The χ curves are narrow peaks, with FWHM increasing with thickness from 1.05° to 1.60°. The m-(−111) reflection, more elongated along χ than the o-(111) spot, is also clearly observed in the frames of the two thickest films of the series.

**Figure 2:**
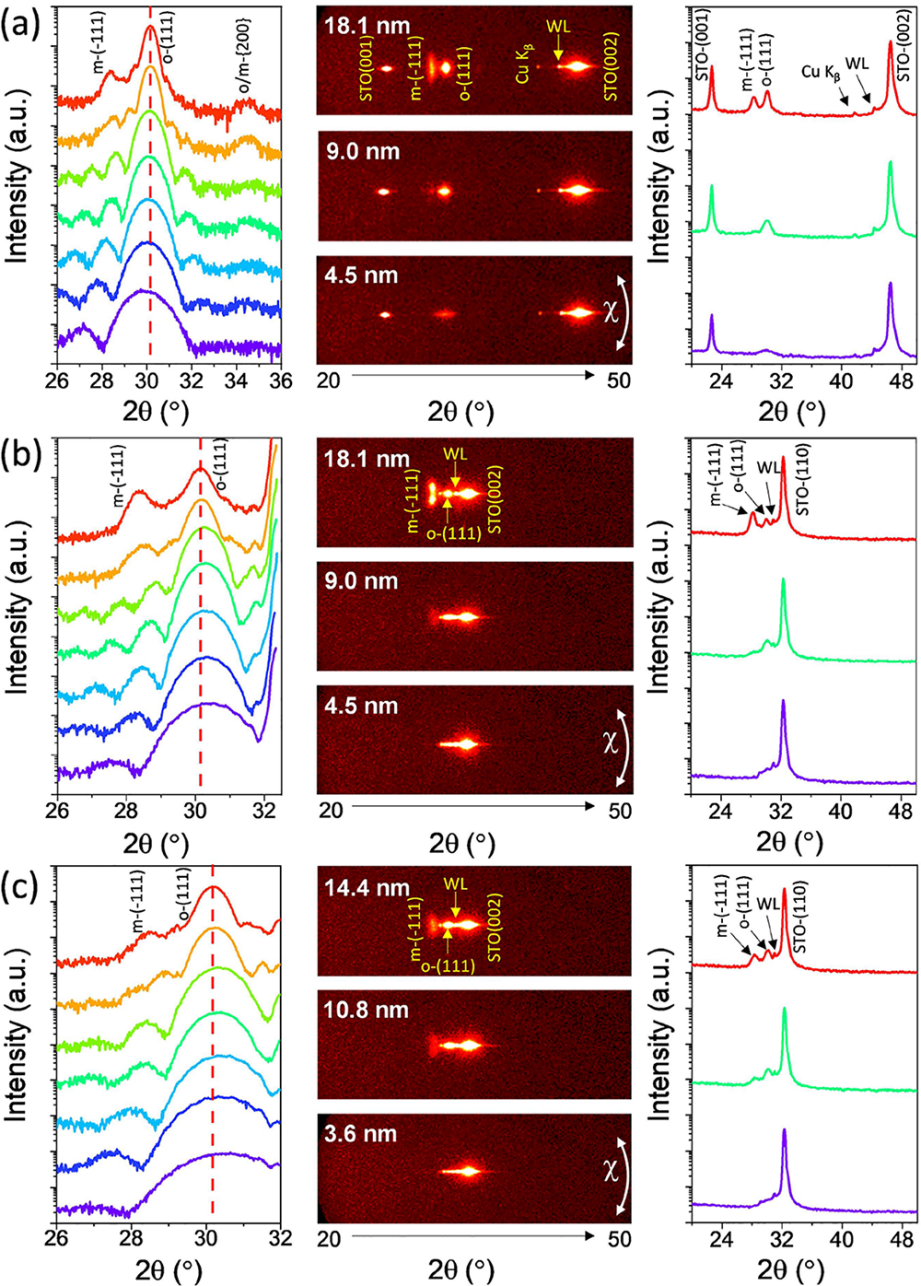
(a) XRD θ-2θ scans measured with point detector detector (left panel), 2θ-χ diffraction frames of films of the indicated thickness (middle panels) with θ-2θ integrated patterns with intensity in the logarithmic scale (right panels) of films on STO(001) deposited under pure O_2_ (series A). (b,c) Same measurements corresponding to films deposited on STO(110) under pure O_2_ (series B) and films on STO(110) under mixed O_2_/Ar, respectively.

Figure 2b shows the XRD diffractograms of the equivalent films deposited under a pure oxygen atmosphere on an STO(110) (series B). The o-(111) peak and accompanying Laue fringes are present in all films and, similar to the films on STO(001), the m-(−111) peak is obvious in the two thickest. The intense STO(110) reflection at around 32.5° (see the 2θ-χ images) is superimposed on the position of the m/o-{200} reflections.^40^ The thickness of the films, obtained by the Laue simulation (Figure S2), matches that of the corresponding films deposited in the same PLD process on STO(001). The position of the o-(111) peak also depends on the thickness, but unlike the films on STO(001), the peak moves toward a smaller angle with increasing thickness, thus indicating a progressive increase in *d*_o‑(111)_. The 2θ-χ diffraction frames (see selected images in Figure 2b and all images in Figure S3) show, similarly to the corresponding frames from the films on STO(001), circularly shaped o-(111) spots in all films and more elongated m-(−111) spots in the thicker films. The width of the χ-curves (Figure S4) also increases with the thickness, from a FWHM of 0.87° (*t* = 4.5 nm) to 1.56° (*t* = 18.1 nm), with the curves being narrower than those of equivalent films on STO(001).

Figure 2c shows the equivalent XRD measurements for the films deposited under a mixed O_2_/Ar atmosphere on an STO(110) (series C). Similar to the films deposited under pure O_2_ (series A and B), the main reflection of the HZO film is the o-(111) and is accompanied by Laue fringes. The m-(−111) peak is also observed in the thickest films. The thickness of the films is smaller than that of the films deposited under pure oxygen with the same number of laser pulses (Figure S5). The lower growth rate per laser pulse is due to a greater scattering of the atoms in the PLD plasma under O_2_/Ar than under pure O_2_ (the total gas pressure is the same, 0.1 mbar, but argon has a mass greater than that of oxygen). The position of the o-(111) peak in this series is also thickness dependent, and the dependence is the same (*d*_o‑(111)_ increases with thickness) as that in films grown on STO(110) under pure oxygen. The 2θ-χ diffraction frames (Figures 2c and S3) are very similar to the equivalent films on STO(110) deposited under a pure oxygen atmosphere. Figure S4 shows that the width of the χ curves increases with thickness in all three series and that for fixed thickness, the width depends on the orientation of the substrate but not on the oxidation conditions (pure O_2_ or mixed O_2_/Ar) during PLD growth. The area of the χ-curves around the o-(111) and m-(−111) reflections of the three series and the o/(o + m) peak areas ratio are plotted as a function of thickness in Figure 3a,b, respectively. The plots clearly show that below a thickness of approximately 10 nm, the presence of the monoclinic phase is minimal, but that it increases rapidly for greater thicknesses, particularly in the films on STO(110). Nevertheless, it should be noted that the graphs correspond to measurements of the peak areas and not to the volume of the Bragg spots in reciprocal space. The mosaicity depends on thickness and substrate orientation, and therefore, the graphs give information on the evolution of the phases with thickness, but do not correspond to the exact quantification of phase content.

**Figure 3:**
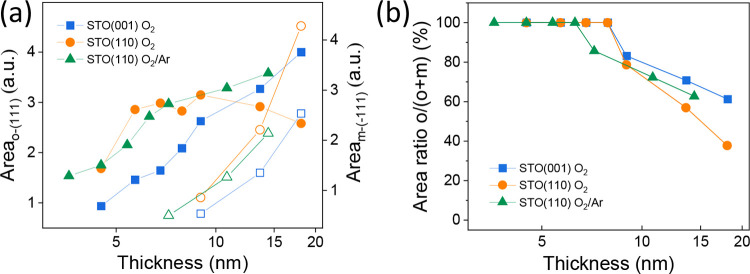
(a) Area of the χ-curves (shown in Figure S4) around the o-(111) (solid symbols) and m-(−111) (empty symbols) reflections as a function of the thickness for series A (blue squares), series B (orange circles) and series C (green triangles). (b) Thickness dependence of o/(o + m) films of series A (blue squares), series B (orange circles) and series C (green triangles).

The dependence of *d*_o‑(111)_ on thickness for the three series is presented in Figure 4a. The lattice parameter of the films on STO(001) decreases progressively with thickness from approximately 2.985 Å (*t* = 4.5 nm film) to a constant value of approximately 2.965 Å in films thicker than about 10 nm. Similar dependence was reported for epitaxial HZO,^41^ La-doped HZO,^42^ and La-doped HfO_2_ ^43^ films on STO(001). The thickness dependence of the films on STO(110) is not influenced by the atmosphere during PLD growth but differs from that of films on STO(001). The thinnest films have the lowest lattice parameter (*d*_o‑(111)_ = 2.93 Å for the *t* = 3.6 nm film), and *d*_o‑(111)_ increases with thickness up to about *t* = 10 nm. The thickest films, for all three series, have a similar lattice parameter of approximately 2.96 Å. The results are in agreement with previous observations of lower *d*_o‑(111)_ in very thin HZO films on STO(110) than in equivalent films on STO(001),^28^ and an increase of *d*_o‑(111)_ with thickness in epitaxial HZO^27^ and La-doped HfO_2_ ^29^ films on STO(110). It should be noted that the o-HZO(111) diffraction peak is close to the intense STO(110) peak, and the partial overlap of the peaks could shift the maximum of the o-HZO(111) peak of the thinner films to a larger angle, causing an underestimation of *d*_o‑(111)_. To evaluate this possibility, the *t* = 4.5 nm film on STO(110) deposited under pure O_2_ was measured by high-resolution XRD using monochromatized Cu K_α1_ radiation (Figure S6). It is seen that the underestimation of *d*_o‑(111)_ for this extremally thin film is very small, confirming that the orientation of the substrate has a critical effect on the thickness dependence of *d*_o‑(111)_, as shown in Figure 4b.

**Figure 4:**
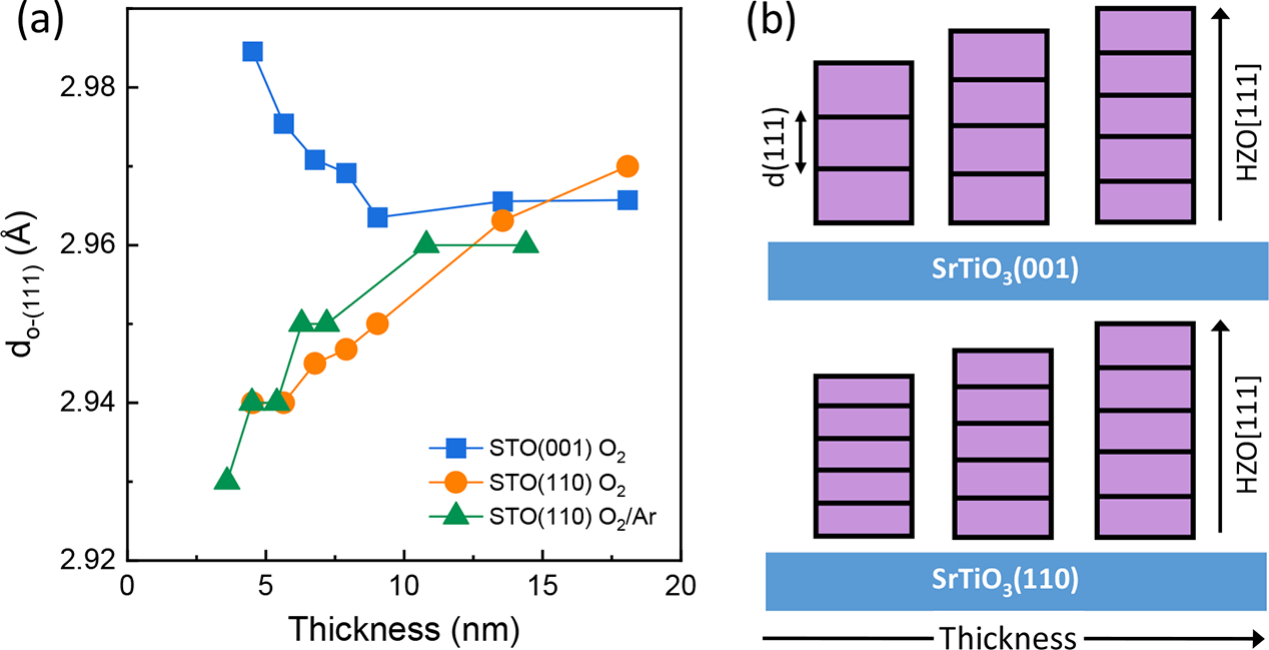
(a) Dependence on the thickness of the out-of-plane lattice parameter of the orthorhombic phase (*d*_o‑(111)_) of films of series A (blue squares), series B (orange circles), and series C (green triangles). (b) Schematics of the evolution with thickness of the out-of-plane lattice parameter of orthorhombic HZO(111) on STO(001) and STO(110).

The lattice mismatch between HZO and LSMO is enormous and does not allow for conventional epitaxial growth. HZO grows (111)-oriented on LSMO(001) and LSMO(110) by the so-called domain matching epitaxy mechanism.^38,44,45^ In conventional epitaxy, there is fully coherent growth below a critical thickness, and thicker films relax, usually through dislocations.^46^ In domain matching epitaxy,^44,45^ the film nucleates by directly forming a semicoherent interface between domains with M lattice planes of the film on N (=M ± 1) planes of the substrate, and having an extra or missing lattice plane. The unconventional epitaxy mechanism and (111) orientation of HZO likely minimize interface and surface energies and allow epitaxial stabilization of the orthorhombic phase. Domains may be under compressive or tensile stress, and different domains may coexist, resulting in an overall minimization of stress. In the case of heteroepitaxy with symmetry continuity at the interface, domain epitaxy would efficiently eliminate the overall stress (excluding stress originating from differences in thermal expansion coefficients). However, heteroepitaxy of the 3-fold symmetry HZO(111) surface on the 4-fold symmetry LSMO(001) or the 2-fold symmetry LSMO(110) surface causes a highly anisotropic stress distribution. This is because a particular ratio of M/N domains can cancel the overall stress in a crystal direction but not in other directions, and the residual stress will induce elastic strain if the film thickness is low. Figure 4a shows the existence of strain for thicknesses less than approximately 10 nm and the fact that the out-of-plane lattice strain is tensile for STO(001) films and compressive for STO(110) films. This indicates that the distinct interface symmetry mismatch depending on the substrate orientation has a critical impact on the lattice strain.

The polarization-electric field loops of the films deposited under pure O_2_ on STO(001) and STO(110), and under mixed O_2_/Ar on STO(110) are shown in Figure 5a–c, respectively. The loops were measured in the pristine state, and the corresponding current-electric field loops are presented in Figure S7. All films are ferroelectric, but the remanent polarization depends on the thickness, substrate orientation, and deposition atmosphere (Figure 6a). Note also that the current-electric field curves (Figure S7) corresponding to the thinnest films of the series B and C show a double current peak, indicating antiferroelectric-like behavior in these two films (the double peak disappears after only one additional cycle). In all three series, *P*_r_ increases first with thickness, has maximum values in the thickness range of 6–10 nm, and decreases in thicker films. The lower polarization measured in the ultrathin films is probably due to their higher leakage (Figure S8), which prevents obtaining completely saturated polarization loops. In the case of the thickest films, there is a drop in polarization related to the presence of a significant amount of monoclinic phase, which is common in both polycrystalline and epitaxial thick films and indicates the importance of the surface energy contribution in stabilizing the metastable orthorhombic phase.^2,23,37^ The lower content of orthorhombic phase reduces the polarization, and, furthermore, a significantly high monoclinic phase fraction can induce depolarizing fields in neighboring orthorhombic grains, leading to an even greater reduction in polarization.^47^ For intermediate thicknesses, in the series of films on STO(001), which was deposited under pure O_2_, the largest *P*_r_ is about 20 μC/cm^2^, while in the series on STO(110) deposited under O_2_ and mixed O_2_/Ar it is about 25 and 35 μC/cm^2^, respectively. Considering that the calculated polarization of orthorhombic hafnia is 52–55 μC/cm^2^ ^33,34^ and that the films are (111)-oriented, the expected maximum polarization in the (111)-oriented films is approximately 32 μC/cm^2^. This is consistent with the measured maximum polarization of 35 μC/cm^2^, which may include a slight overestimation due to the current leakage contribution. The coercive electric field (*E*_C_) is also thickness dependent in all three series, with a decrease of *E*_C_ increasing thickness for films thicker than ∼5 nm (Figure 6b). The experimentally lower *E*_C_ in films thinner than 5 nm (open symbols) may be due to polarization undersaturation due to the ultralow thickness, which prevents the application of a larger electric field, resulting in an underestimation of *E*_C_. For thicker films, the *E*_C_ thickness dependence closely follows the Janovec–Kay–Dunn^48,49^ (*E*_C_ ∝ *t* ^–2/3^) scaling (see the magenta line) characteristic of high-quality ferroelectrics, already observed in epitaxial hafnia films but elusive in polycrystalline samples.^37^

**Figure 5:**
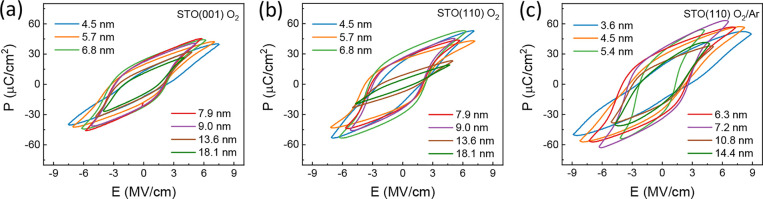
Ferroelectric polarization loops of (a) films on STO(001) deposited under pure O_2_ (series A), (b) films deposited on STO(110) under pure O_2_ (series B), and (c) films on STO(110) under mixed O_2_/Ar.

**Figure 6:**
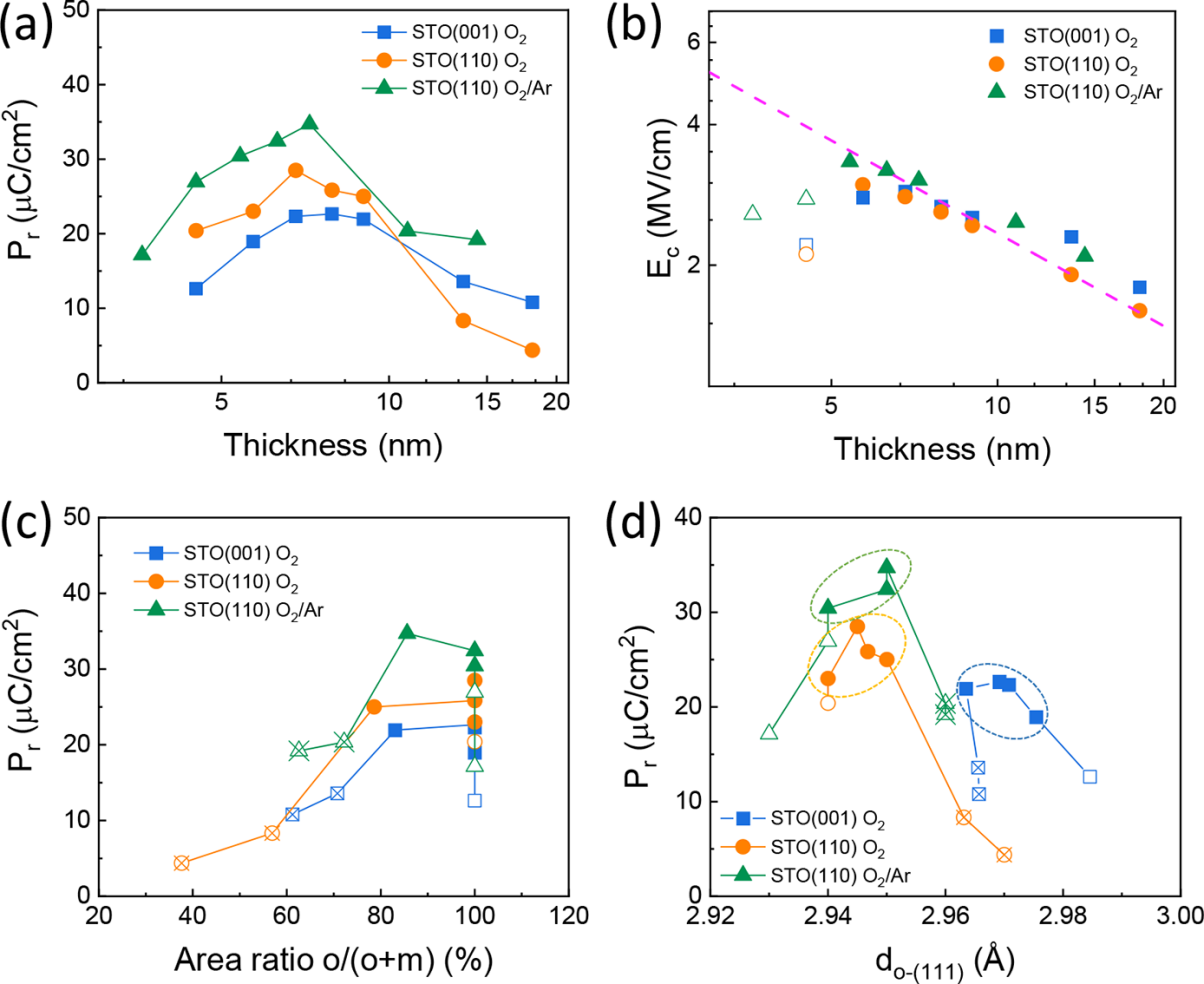
(a) Dependence on the thickness of the remanent polarization of films of series A (blue squares), series B (orange circles) and series C (green triangles). (b) Dependence on the thickness of the electric coercive field. The magenta line (slope −0.67) is a guide to the eye to compare the experimental dependence with the *E*_C_ ∝ *t* ^–2/3^ scaling. The films thinner than 5 nm (open symbols) deviate of this dependence. (c) Remanent polarization as a function of the o/(o + m) fraction of peaks areas in XRD χ curves (Figure 3). (d) Remanent polarization plotted as a function of the out-of-plane lattice parameter of the orthorhombic phase (*d*_o‑(111)_). In (c) and (d): open symbols correspond to films thinner than 5 nm, which deviate from the Janovec–Kay–Dunn dependence in (b). Open symbols with a cross correspond to films thicker than 10 nm, which contain a high amount of the nonferroelectric phase that can create depolarization fields.

The thickness dependence of the polarization in the three series (Figure 6a) shows that for the same thickness, the polarization is greater in films on STO(110) than on STO(001), and greater in films deposited under reducing conditions. Only the thickest films grown on STO(110) under pure O_2_, which exhibit strong polarization reduction, deviate from this behavior. The remanent polarization is plotted as a function of the o/(o + m) fraction of peak areas in XRD χ curves in Figure 6c. The graph shows that when the o/(o + m) fraction decreases below approximately 75% (open symbols with a cross), the polarization decreases noticeably, suggesting that the high amount of monoclinic phase in these films causes depolarizing fields that suppress the polarization of part of the existing orthorhombic grains.^47^ This increased content of monoclinic phase occurs, in all three series, in the films with thicknesses of >10 nm (see values in Table S2). We note that most ferroelectric hafnia-based memory devices require films less than 10 nm thick, and therefore, the polarization drop observed here for films thicker than about 10 nm is not a limitation. In the thinner films in each series, the polarization is probably influenced by the presence of high leakage. The films with loops that were more undersaturated are likely those that clearly deviate from the Janovec–Kay–Dunn dependence in the *E*_C_–thickness graph (open symbols). Thus, we focus on the films with intermediate thickness (solid symbols) in each series to evaluate the impact of interface symmetry, redox conditions, and strain on the polarization. To distinguish between the impact of interface symmetry, the oxidation conditions, and the strain, the remanent polarization of the films of the three series is plotted in Figure 6d as a function of *d*_o‑(111)_. Comparing the three sets of data, there is no correlation between polarization and strain. The films on STO(001) have *d*_o‑(111)_ values in the 2.963–2.975 Å range and the same polarization of 19–23 μC/cm^2^. In the case of films on STO(110) deposited under pure O_2_, *d*_o‑(111)_ is in the small range of 2.94–2.95 Å, and polarization is approximately 23–28 μC/cm^2^. In the other series on STO(110), the mixed O_2_/Ar does not have an important effect on *d*_o‑(111)_ (being in the 2.94–2.95 Å), while the films present the highest polarization (in the range of 30–35 μC/cm^2^). In contrast, Figure 6d confirms that the interface symmetry and redox conditions are important factors. Comparing the two series deposited under a pure O_2_ atmosphere, films on STO(110) have higher polarization than those on STO(001). Comparing the two series of films deposited on STO(110), we found that films deposited under mixed O_2_/Ar have higher polarization. The polarization is also lowered if the film contains a fraction of nonferroelectric monoclinic phase, and when part of the orthorhombic phase cannot be switched due to the presence of defects that pin the polarization.

## 4 Conclusions

In conclusion, the selection of the orientation of the substrate, reducing conditions, and the thickness of the film is crucial in the stabilization of the ferroelectric phase and the polarization of the films. Polarization is enhanced in films on STO(110) instead of the more commonly used STO(001), and in films deposited under a mixed O_2_/Ar atmosphere instead of the usual atmosphere of pure oxygen. Substrate orientation, oxidation conditions, and film thickness can be varied as parallel parameters that influence the relative formation energy of the hafnia polymorphs and can be synergistically adjusted to obtain films almost free of nonferroelectric phases with the maximum expected polarization for orthorhombic phase pure films. We also conclude that strain is not a major factor affecting the formation of the metastable ferroelectric phase neither polarization.

## Supplementary Material



## Notes

Table summarizing thickness and deposition parameters of the investigated samples; full-width at half-maximum (FWHM) along 2θ of o-HZO(111); simulation of Laue oscillations; XRD 2θ-χ frames; XRD χ-curves; evolution with thickness of XRD diffraction peaks; thickness of HZO films; XRD θ-2θ scan measured with monochromatized Cu Kα_1_ radiation; current – electric field curves; leakage current; and table summarizing thickness, XRD area peaks ratio, *d*_o‑(111)_ lattice parameter, leakage, remanent polarization, and coercive electric field of the investigated samples (PDF)
