# Supplementary material for: Cooperative Effects of Interface Symmetry, Redox Conditions and Low-Thickness to Improve Polarization in Ferroelectric Hf0.5Zr0.5O2 Films
Source: ACS Appl Mater Interfaces. 2025 May 26;17(22):32596–603. doi: 10.1021/acsami.5c03527 (PMC12147076; doi:10.1021/acsami.5c03527)
Supplement: Supplementary file 1 [file am5c03527_si_001.pdf]

Supporting Information

Cooperative effects of interface symmetry, redox conditions and low-thickness to improve polarization in ferroelectric $Hf_{0.5}Zr_{0.5}O_2$ films

Xueliang Lyu, Faizan Ali, Tingfeng Song, Ignasi Fina* and Florencio Sánchez*

Institut de Ciència de Materials de Barcelona (ICMAB-CSIC), Campus UAB, Bellaterra 08193, Barcelona, Spain.

* Corresponding authors: ifina@icmab.es (Ignasi Fina) and fsanchez@icmab.es (Florencio Sánchez)

Table S1: Table summarizing investigated samples

Series	A	B	C
**Substrate**	$SrTiO_3$(001)	$SrTiO_3$(110)	$SrTiO_3$(110)
**Atmosphere**	O_2	O_2	O_2/Ar
**Thickness (nm)**	4.5	4.5	3.6
	5.7	5.7	4.5
	6.8	6.8	5.4
	7.9	7.9	6.3
	9.0	9.0	7.2
	13.6	13.6	10.8
	18.1	18.1	14.4

Table S1: Substrate, gas atmosphere during growth of HZO, and HZO thickness of samples of series A, B and C.

Figure S1: Full-width at half-maximum (FWHM) along 2θ of o-HZO(111)

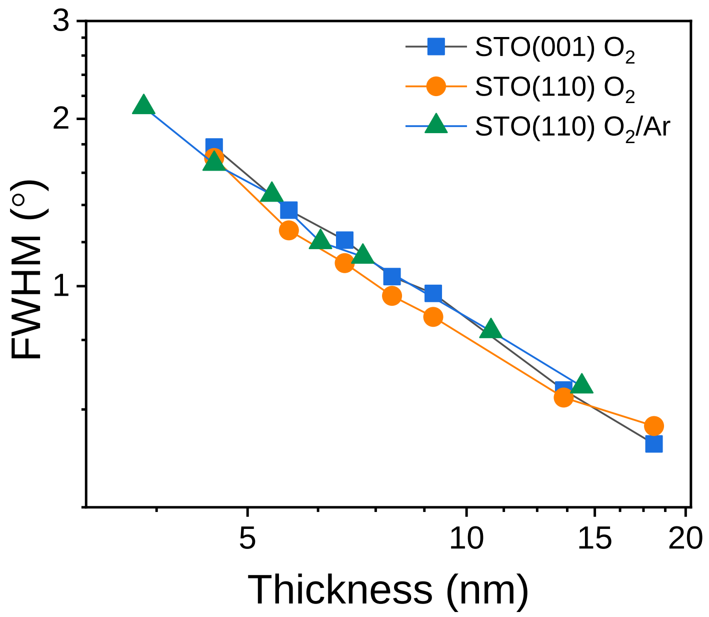

Figure S1: Dependence on the thickness of the full-width at half-maximum (FWHM) along 2θ of the orthorhombic (111) diffraction peak of films of series A (blue squares), series B (orange circles) and series C (green triangles).

Figure S2: Simulation of Laue oscillations

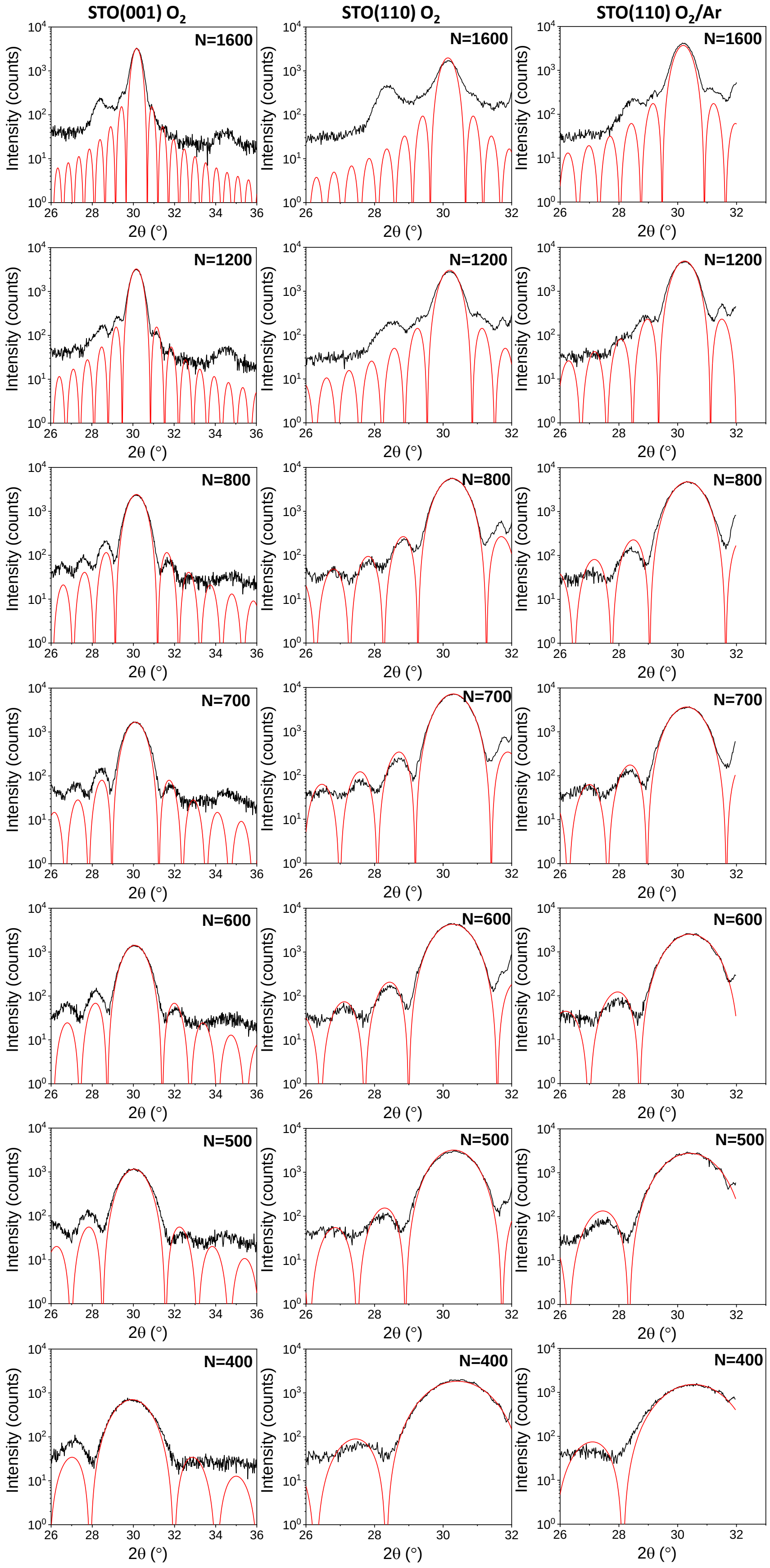

Figure S2: XRD θ-2θ scans around the o-HZO(111) reflection of films of series A (left), series B (middle) and series C (right). The number of laser pulses used to deposit each film is indicated in the corresponding scans. Scans are vertically according to the increased number of pulses, being placed in the bottom corresponding to the films deposited with the lower number of pulses (400). The o-HZO(111) reflection is simulated in each panel (red curve) according to the equation:

$$I(Q) = \left(\frac{\sin\left(\frac{QNc}{2}\right)}{\sin\left(\frac{Qc}{2}\right)} \right)^2$$

where Q = 4πsin(θ)/λ is the reciprocal space vector, N the number of unit cells along the out-of-plane direction and c the corresponding lattice parameter.

Figure S3: XRD 2θ-χ frames

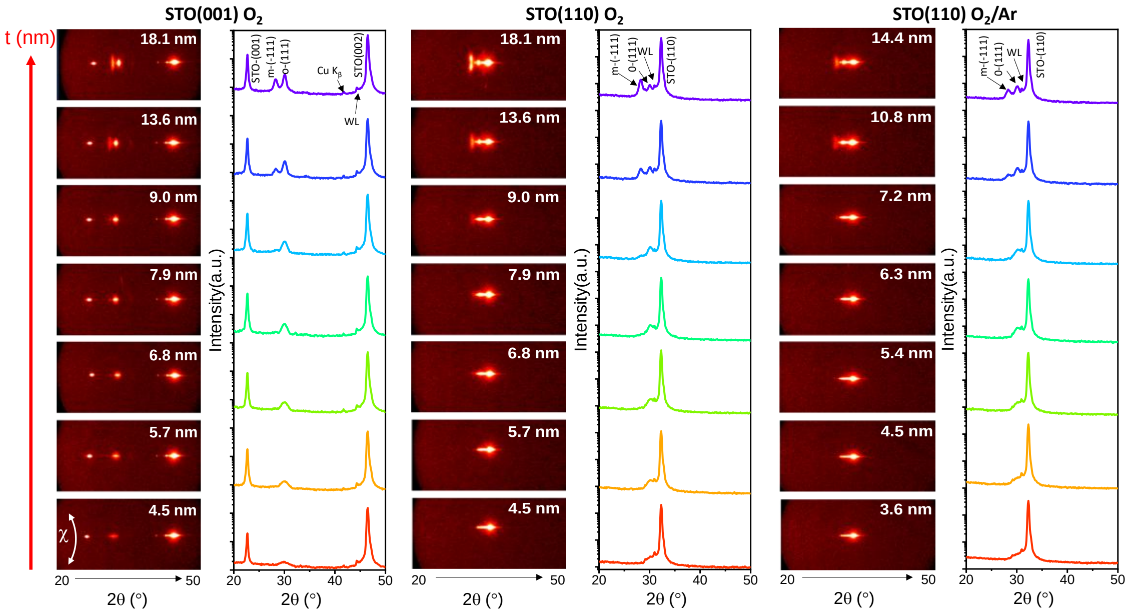

Figure S3: Left panels: XRD 2θ-χ frames of films on STO(001) deposited under pure O_2 (series A) and θ-2θ integrated patterns with intensity in logarithmic scale. The film thickness is indicated in the corresponding pattern. The equivalent 2θ-χ diffraction frames and θ-2θ integrated patterns corresponding to films deposited on STO(110) under pure O_2 (series B) and films on STO(110) under mixed O_2/Ar are shown in the middle and right panels, respectively.

Figure S4: XRD χ-curves

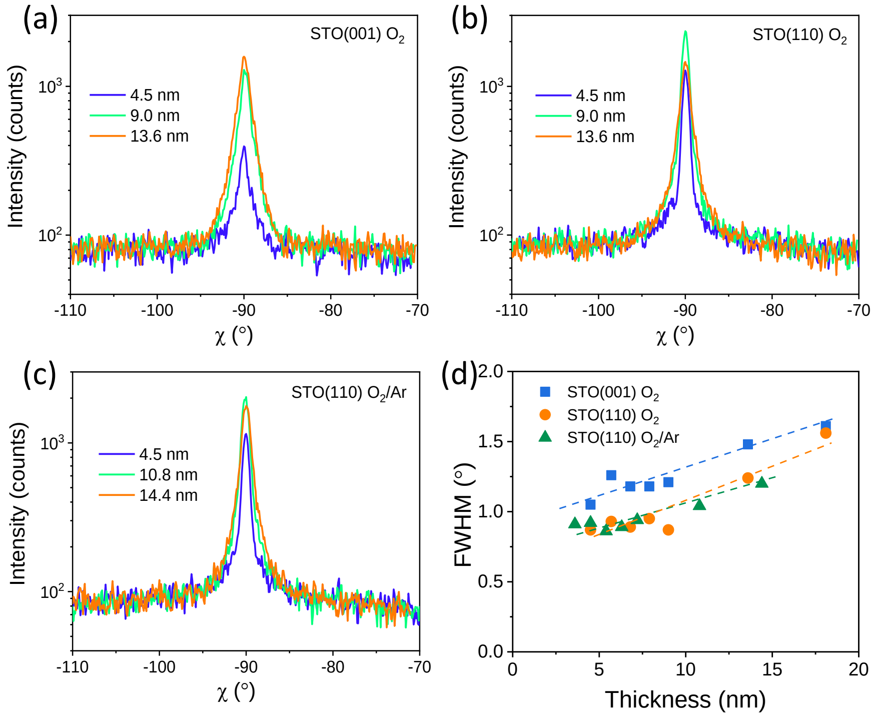

Figure S4: XRD χ-curves of selected films of the indicated thickness of (a) series A, (b) series B, and (c) series C. The χ-curves were obtained by integration of 2θ-χ images in a 2θ range ±0.5° around the intensity maximum. (d) Dependence on the thickness of the full-width at half-maximum (FWHM) along χ of the orthorhombic (111) diffraction peak of films of series A (blue squares), series B (orange circles) and series C (green triangles).

Figure S5: Thickness of HZO films

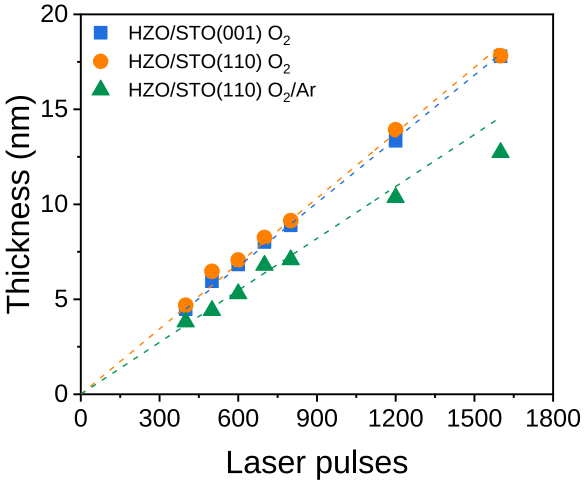

Figure S5: Thickness of films of series A (blue squares), series B (orange circles) and series C (green triangles) as a function of the number of laser pulses in the corresponding PLD process.

Figure S6: XRD θ-2θ scan measured with monochromatized Cu Kα$_1$ radiation

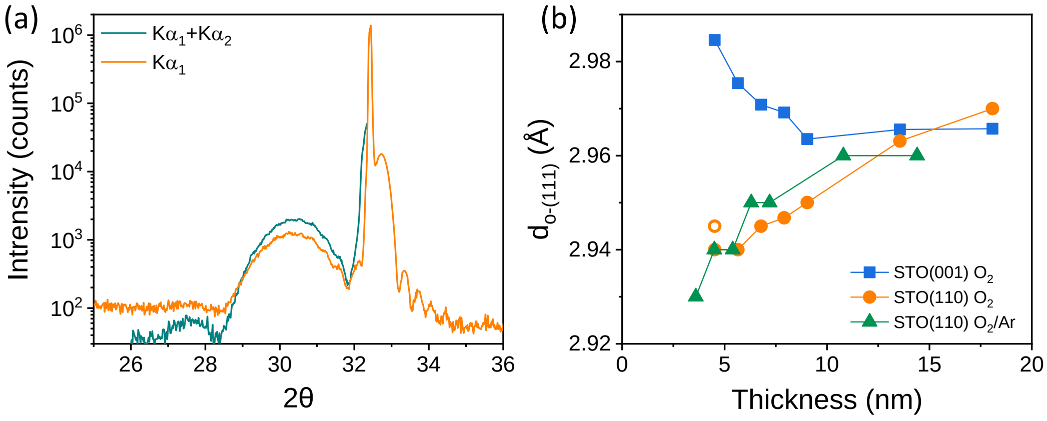

Figure S6: (a) XRD θ-2θ scans of the t = 4.5 nm film deposited on STO(110) under pure O_2, measured with a standard diffractometer and Cu Kα$_1$ and Cu Kα$_2$ radiation (green line) and measured with high resolution diffractometer and monochromatized Cu Kα$_1$ radiation (orange line). (b) Dependence of $d_{o\text{-}(111)}$ on thickness shown in Figure 3. Open orange circle: $d_{o\text{-}(111)}$ of the t = 4.5 nm film deposited on STO(110) under pure O_2, measured with monochromatized Cu Kα$_1$ radiation.

Figure S7: Current – electric field curves

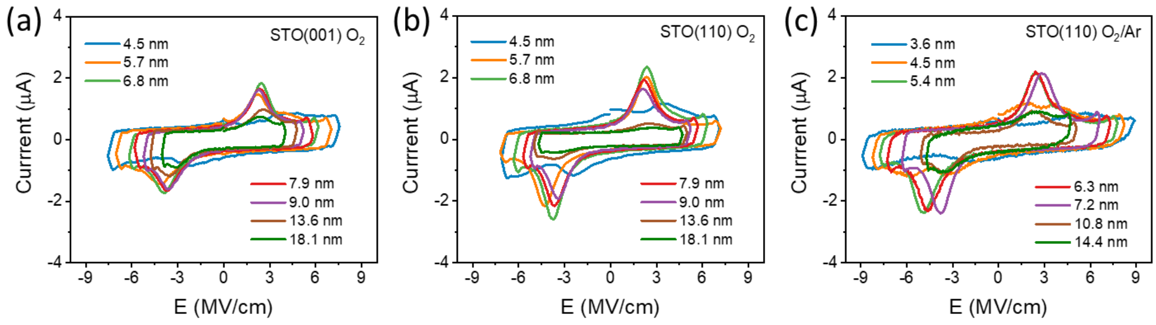

Figure S7: Current – electric field curves corresponding to the ferroelectric polarization loops shown in Figure 5 for (a) films on STO(001) deposited under pure O_2 (series A), (b) films deposited on STO(110) under pure O_2 (series B), and (c) films on STO(110) under mixed O_2/Ar.

Figure S8: Leakage current

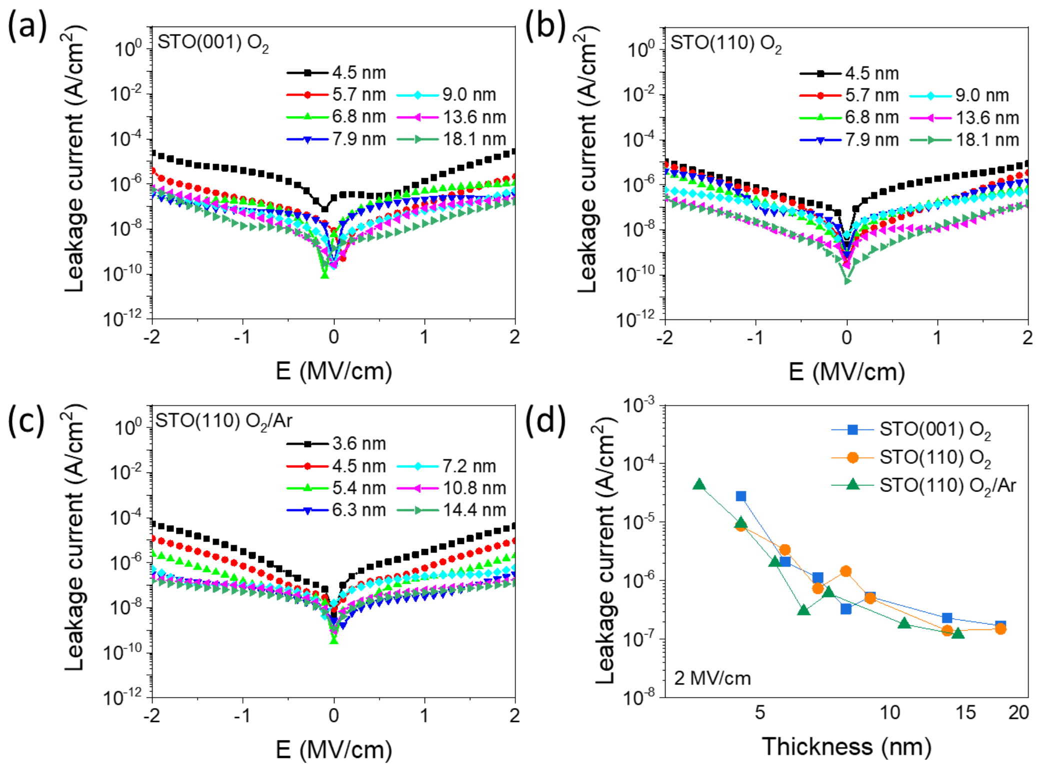

Figure S8. Current – electric field curves measured in direct current conditions of (a) the set of films deposited on STO(001) under pure O_2 (series A), (b) the set of films deposited on STO(110) under pure O_2 (series B), and (c) the set of films deposited on STO(110) under mixed O_2/Ar. (d) Thickness dependence of the leakage current density at 2 MV/cm for the three series.

Table S2: Table summarizing investigated samples

Series A: $SrTiO_3(001)$, O_2

Thickness (nm)	Area ratio o/(o+m)	$d_{o\text{-}(111)}$ (Å)	Leakage @ 2MV/cm (μA/cm²)	P_r (μC/cm²)	E_c (MV/cm)
4.5	100%	2.984	28	12.7	2.2
5.7	100%	2.975	2.1	18.9	2.8
6.8	100%	2971	1.1	22.3	2.9
7.9	100%	2.969	0.33	22.7	2.7
9.0	83.1%	2.964	0.53	21.9	2.5
13.6	70.8%	2.966	0.53	13.6	2.3
18.1	61.2%	2.966	0.17	10.8	1.8

Series B: $SrTiO_3(110)$, O_2

Thickness (nm)	Area ratio o/(o+m)	$d_{o\text{-}(111)}$ (Å)	Leakage @ 2MV/cm (μA/cm²)	P_r (μC/cm²)	E_c (MV/cm)
4.5	100%	2.940	8.68	20.4	2.11
5.7	100%	2.940	3.39	23	2.97
6.8	100%	2.945	0.74	28.5	2.8
7.9	100%	2.947	1.45	25.8	2.6
9.0	78.6%	2.950	0.5	25	2.43
13.6	56.9%	2.963	0.14	8.3	1.91
18.1	37.6%	2.970	0.15	4.4	1.6

Series C: $SrTiO_3(110)$, O_2/Ar

Thickness (nm)	Area ratio o/(o+m)	$d_{o\text{-}(111)}$ (Å)	Leakage @ 2MV/cm (μA/cm²)	P_r (μC/cm²)	E_c (MV/cm)
3.6	100%	2.927	42.55	17.2	2.57
4.5	100%	2.939	9.49	27	2.77
5.4	100%	2.938	2.01	30.4	3.33
6.3	100%	2.947	0.3	32.4	3.19
7.2	85.6%	2.945	0.61	34.7	3.04
10.8	72.2%	2.956	0.18	20.4	2.47
14.4	62.6%	2.960	0.12	19.2	2.09

Table S2: Summary of thickness, o/(o+m) area ratio χ-curves (shown in Figure S4), $d_{o\text{-}(111)}$, leakage, remanent polarization (P_r) and coercive electric field (E_C) for samples of series A, B and C.
